# Health professionals' overestimation of knowledge on snakebite management, a threat to the survival of snakebite victims—A cross-sectional study in Ghana

**DOI:** 10.1371/journal.pntd.0008756

**Published:** 2021-01-19

**Authors:** Evans Paul Kwame Ameade, Isaac Bonney, Evans Twumasi Boateng

**Affiliations:** 1 Department of Pharmacy, School of Medicine and Health Science, University for Development Studies, Tamale; 2 South Tongu District Hospital, Sogakofe, Ghana; 3 Worawora Government Hospital, Worawora, Ghana; Watamu Hospital, KENYA

## Abstract

**Background:**

According to the World Health Organization, snakebites, a common occupational hazard in developing countries accounts for an annual loss of between 81,000 and 138, 000 lives following 5 million bites of which 2.7 million results in envenomation. Since snakebite-associated morbidity and mortality are more prevalent in agricultural economies such as Ghana, health professionals should be optimally knowledgeable on how to manage the incidence of snakebites. Lack of knowledge or overestimation of a professional's knowledge can affect heath delivery especially for emergencies such as snakebites. The three rurally situated Tongu districts in South-Eastern Ghana with agriculture as the major source of livelihood for their inhabitants, are prone to snakebites. This study, therefore, brings up the need to assess whether the health professionals in these districts are well-equipped knowledge-wise to handle such emergencies and whether they can rightly estimate their knowledge with regards to snakebite management.

**Methodology/Principal findings:**

Data was collected using a *de novo* semi-structured questionnaire administered through google form whose link was sent via WhatsApp to 186 health workers made up of nurses, midwives, physician assistants, medical doctors, pharmacists, and pharmacy technicians. This data was analyzed using Statistical Package for the Social Sciences (SPSS) Version 25. Association between variables was determined using the appropriate tools where necessary, using a confidence interval of 95% and significance assumed when p ≤ 0.05. This study found male health workers significantly more knowledgeable about snakebite management (11.53±5.67 vs 9.64±5.46; p = 0.022) but it was the females who overestimated their knowledge level (27.9% vs 24.1%). The medical doctors exhibited the best knowledge on snakebite management with the registered general nurses least knowledgeable. Although most professionals overestimated their knowledge, the registered general nurses were the worst at that (53.7%). Overall knowledge of health care professionals on snakebite management was below average [10.60±5.62/22 (48.2%)] but previous in-service training and involvement in the management of snakebite were associated with better knowledge. Respondents who had no previous training overestimated their knowledge level compared to those who had some post-qualification training on snakebite management (38.1% vs 7.5%). The greatest knowledge deficit of respondents was on the management of ASV associated adverse reactions.

**Conclusion:**

Health workers in rural Ghana overestimated their knowledge about snakebite management although their knowledge was low. Training schools, therefore, need to incorporate snakebite management in their curriculum and health authorities should also expose health workers to more in-service training on this neglected tropical disease.

## Author summary

World Health Organization estimates that every year between 81,000 and 138,000 die due to snake bites across the world. Mismanagement of snakebites can result in increased disabilities and death if not handled by knowledgeable health workers. This study assessed if various categories of health workers made up of professionals from the medical, pharmaceutical, and nursing categories in the three neighbouring Tongu districts in Ghana have the appropriate level of knowledge on snakebite management. Using a newly developed questionnaire, data were collected from the respondents using google forms sent to their WhatsApp platforms. Data were then analyzed using Statistical Package for the Social Sciences (SPSS) Version 25. Results were presented in the form of tables and association between the variables also determined. The level of knowledge of sampled health workers on snakebite was below average, especially among nursing professionals. However, those who had some previous post-qualification training on snakebite management exhibited a significant superior knowledge and least overestimated their knowledge hence policymakers should through workshops equip health workers especially the nurses on snakebites so that rural dwellers whose health care needs are mainly attended to by nurses can be better managed when they suffer snakebites.

## Introduction

Snakes which belong to the class of animals called the reptiles can be found in all places except in Antarctica, Iceland, Ireland, Greenland, New Zealand, Cape Verde in West Africa, Siberia area in Russia, some parts of Argentina, Chile, Finland as well as some small nations in the Pacific Ocean such as Tuvalu and Nauru [1]. It is estimated that there are more than 3,700 species of snakes on earth [2]. As snakes also make efforts to survive in the ecosystem, there are bound to come into conflict with humans, and mostly as a defensive mechanism some of them bite. This human-snake conflict is estimated to results in between 4.5 and 5.4 million snakebites annually [3]. It is estimated that about 600 snakes whenever they bite, they inject toxins substances referred to as venoms into their victims hence they are classified as being venomous while the vast majority are non-venomous [4]. The number of persons bitten by venomous snakes cannot be exactly known but it is believed that 1.8 to 2.7 million people globally suffer the effects of their bites out of which 81,000 to 138,000 victims die although the mortality would have been higher had it not been because about 50% of venomous snakebites do not lead to envenoming [4,5]. Notwithstanding this high level of snakebite incidence, reports across the world found that quite many victims seek remedies from traditional medicine practitioners than hospitals. A study in India found that only 22.2% of snakebite victims report at the hospitals [6]. Two hospital-based surveys in Nigeria and Ghana reported snakebite incidence of 465 per 100,000 and 92 per 100,00 respectively [7,8]. Mortality and morbidity associated with snakebites for those who report at the hospital can be determined by the level of management by the health care professionals which will depend on how knowledgeable or skillful they are on snakebite management. Since there is a paucity of study on the assessment of the knowledge of healthcare professionals on the management of snakebites in Ghana, this study assessed if various categories of health workers made up of professionals from the medical, pharmaceutical and nursing categories in the three neighbouring Tongu districts in the coastal savanna ecozone of Ghana have the appropriate level of knowledge on snakebite management.

## Method

### Ethics statement

The ethics committee of the School of Medicine and Health Sciences of the University for Development Studies, Tamale granted ethical clearance for this study. Again, the preamble on the questionnaire explained the purpose of the research and stating clearly that submitting the form after completion was indicative of giving consent. To ensure confidentiality, the names of the respondents were not required. Clearances were also obtained from the District Health Directorates as well as the administrative heads of the various health facilities from which the data were collected.

### Study setting

**Fig 1:**
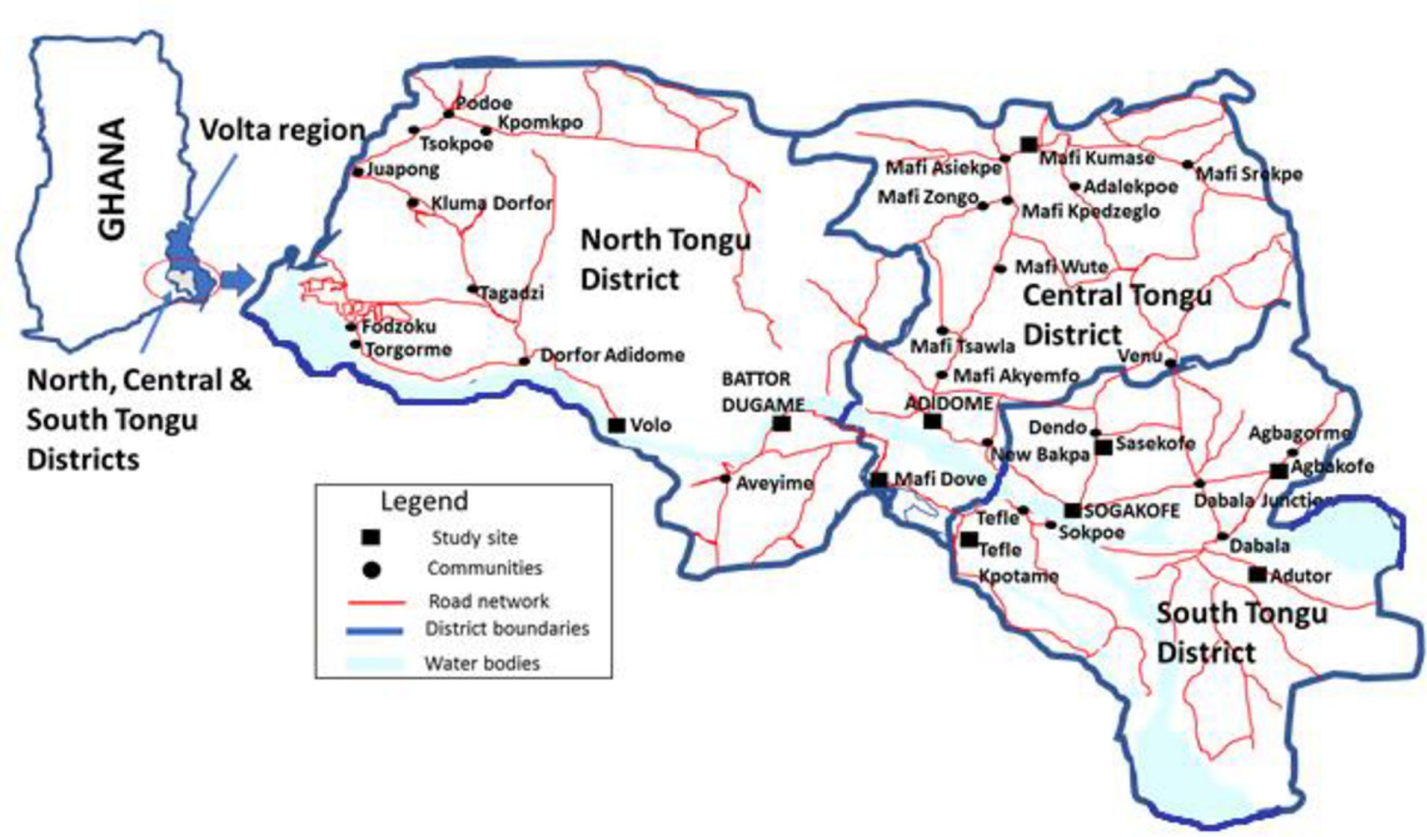
The map of Ghana and the study location, the South, Central, and Tongu Districts of the Volta region of Ghana.

The study areas are selected health facilities in the South, Central, and North Tongu districts of the Volta region of Ghana. The facilities in the South Tongu district were the District Hospital and Comboni Catholic Hospital both located at Sogakofe; Health Centres at Tefle Kpotame and Adutor and the Agbakofe and Sasekofe Community-based Health Planning and Service (CHPS) zones. CHPS zones are the lowest level of the health care system in Ghana for the provision of primary health care to those in rural Ghana. For the North Tongu District, Battor Catholic Hospital and Volo Health Centre were the sites for the study while the Central Tongu District had the District Hospital at Mafi Adidome as well as Mafi Kumase and Mafi Dove Health Centres as the study sites. The total population of these three Tongu districts in Ghana's 2010 National population census was 237,138 [9]. Inhabitants of these districts (Fig 1) whose main occupation is agriculture-related, speak mainly the Tongu dialect of the Ewe language.

### Study design

A cross-sectional study design was applied in this study which was conducted within May and June 2019.

### Study population

The study population was health care providers namely; pharmacists, physician/medical assistants, medical doctors, pharmacy technicians as well as midwives, and nurses of various categories who work in hospitals, health centres, and CHPS compounds in the study area.

### Study sample size determination

The sample size for this study was calculated using the Cochran formula,

$$n_{0}=\frac{t^{2}(p)(q)}{d^{2}}$$

Where t (selected alpha level of .025 in each tail) = 1.96; d, (acceptable margin of error) = 0.05; With an estimated overall knowledge of health care providers in the study area on the management of snakebites as 50%, p (the estimated proportion of an attribute that is present in the population) = 0.5 hence q is 1-p = 0.5.

$$n_{0}=\frac{1.96^{2}(0.5)(0.5)}{0.05^{2}}$$

$$n_{0}=384$$

Since *n*_0_ *of* 384 exceeds the 5% of the eligible study population of 537 excluding the 20 involved in the pre-testing of the questionnaire (537 x 0.05 = 26.9), the Cochran correction formula can be used to obtain the adjusted sample size *n*_1_

$$n_{1}=\frac{n_{0}}{1+\frac{n_{0}}{Study\;population}}$$

$$n_{1}=\frac{384}{1+\frac{384}{517}}$$

$$n_{1}=220$$

With an expected response rate of 90%, the final actual sample size for the study was 244 ($\frac{n_{1}}{0.9}$).

### Sampling procedure

Efforts were made to take a census sample of all the pharmacists (5), physician/medical assistants (26), medical doctors (17), and pharmacy technicians (6) because of their small numbers in the selected health facilities. However, for the nurses and midwives who were about 483, a convenience sampling technique was applied to select the respondents. For the category of health professionals that census technique was applied, they were met in person or spoken to on their mobile phone and the link of the questionnaire was sent to the WhatsApp pages of those willing to partake in the study. All nurses and midwives working in Health centres and CHPS zones which are the lowest level of health care in Ghana were invited to partake in the study after a visitation by a member of the research team. For the respondents from the hospitals, the invitation was extended to those who were at the facility at the time of the visit of the research team. Some off-duty nurses and midwives were gotten in to participate in the study by their colleagues who the researchers had earlier met and enrolled in the study.

### Data collection instrument and technique

A de novo self-administered semi-structured questionnaire was designed and then converted into a google form. The questions were formulated based on information obtained from the 2016 edition of the WHO Guidelines for the management of snakebites, WHO Regional Office for South-East Asia. The questionnaire was piloted among twenty (20) health workers from the study area who were subsequently excluded during the actual data collection. The research team performed a face validity of the questionnaire and also deleted or reframed questions that were ambiguous to ensure their clarity. The reliability test of the questionnaire was also performed using Microsoft Excel which gave a Cronbach alpha of 0.7 which made the questionnaire acceptable for the study. The questionnaire consisted of thirty-one questions of which six were on respondents' sociodemographic characteristics, and another nine related to previous training and management as well as their level of confidence in the management of snakebites. The remaining fifteen questions assessed respondents' knowledge about snakebite management. The questionnaire was administered through the WhatsApp accounts of the respondents using the link https://forms.gle/iV5NtKzdjbg5LTSc9. Follow up text messages were sent and calls made to the respondents to remind them of the need to complete and submit the questionnaire.

### Data measurement

Comparing the answers provided by the respondents with those from literature sources, the knowledge of the health professionals on snakebite management was assessed. For questions that the respondents had the option of choosing the most appropriate answer, a score of 1 mark was allocated. Choosing a wrong or an 'I don't know' option attracts a zero mark. The total score for open-ended questions depends on the maximum number of answers required to be provided hence a question that required the provision of four answers scores 4 marks if all the respondent's answers are considered appropriate. The total maximum score which assessed the respondent's knowledge on snakebite management was 22. In comparing the perceived and actual knowledge scores of respondents on snakebite management, the total score of actual knowledge of each respondent was converted to 10 because respondents stated their perceived knowledge on snakebite management with values ranging between 0 and 10 with 0 indicating absolute lack of knowledge while 10, for the most excellent level of knowledge.

### Statistical analysis

Descriptive data were presented in tables indicating frequencies and percentages of the variables and questions used for the assessment. Association between variables was also determined using One-Way ANOVA at a confidence interval of 95%. Assessment of the level of over or underestimation or exaggeration of respondents was measured by comparing respondents perceived knowledge and their actual knowledge score using paired sample test and pair sample correlation. Significance was assumed when p ≤ 0.05.

## Results

### Sociodemographic characteristics of respondents

At the end of the study period, responses from 186 individuals out of the expected 244 were successfully received resulting in a response rate of 76.2% (186/244*100). The number of males and females who took part in the study was almost equal (51.1% vs 48.9%) but those between ages 30 and 39 years were in the majority, 98 (52.7%). Again, the majority of respondents were health workers in hospitals, 146 (78.5%), and had worked for less than 5 years, 112 (60.2%). Most respondents were from the South Tongu District, 87 (46.8%), and were registered general nurses, 80 (43.0%) but the health profession least represented were the pharmacists, 4 (2.2%). Table 1 shows the sociodemographic characteristics of respondents in this study.

**Table 1:** Sociodemographic characteristics of respondents.

Variable	Subgroup	Frequency	Percentage
Sex	Male	95	51.1
	Female	91	48.9
Age (years)	20–29	81	43.5
	30–39	98	52.7
	>39	7	3.7
District	South Tongu	87	46.8
	Central Tongu	45	24.2
	North Tongu	54	29
Number of years of practice (years)	< 5	112	60.2
	5–10	71	38.2
	>10	3	1.6
Level of health facility	CHPS zones	15	8.1
	Health Centre	25	13.4
	Hospital	146	78.5
Profession category	Registered General Nurse (RGN)	80	43.0
	Community Health Nurser/Enrolled nurse (CHN/EN)	49	26.3
	Midwife	15	8.1
	Medical officer	14	7.5
	Pharmacy Technician	5	2.7
	Pharmacist	4	2.2
	Physician assistant*	19	10.2

* Physician assistant included nurse practitioners and medical assistants who also consult in Ghanaian health facilities

### Training on and management of snakebite

Table 2 presents the record of post-qualification training on snakebite management and management history of respondents. Although, those who had ever been provided training on snakebite management since they started practicing as healthcare professionals were in the minority, 57 (30.6%), majority of the respondents, 154 (82.8%) had ever been involved in the management of snakebite victims in their facilities. For those who had no post-qualification formal training on snakebite management, most, 53 (40.2%) had snakebite management skills from their senior colleagues with a lesser number, 32 (24.2%) acquiring their knowledge by reading materials from the internet and books. For the first half of the year 2019, most, 92 (49.5%) respondents who had ever managed snakebite cases had taken care of between 1 and 5 victims. Although the majority, 160 (86.0) will triage snakebite as an emergency, most respondents, 91 (48.9%) do not think their health facilities have all the resources for optimal management of snakebites. The major limitation against the management of snakebite for those who think their health facilities cannot manage snakebites adequately is the unavailability of anti-snake venom,77 (86.5%) although the majority of respondents, 139 (73.7%) of all respondent said their health facilities have protocols for the management of snakebites. Most respondents, 79 (43.6%) were fairly confident about their ability to manage snakebite victims.

**Table 2:** Training on and management of snakebite incidence by respondents.

Variable	Subgroup	Frequency	Percentage
Ever managed snakebite	Yes	154	82.8
	No	32	17.2
Ever been trained on snakebite management after school through seminars or workshops?	Yes	57	30.6
	No	129	69.4
If not trained, how was skill acquired? (n = 132)	Learning from senior colleagues on the job	53	40.2
	Knowledge and skills obtained in school	47	35.6
	Self-education on the internet or in textbooks	32	24.2
Number of snakebites managed half-year (January to June 2019)	0	77	41.4
	1–5	92	49.5
	6–10	12	6.5
	>10	5	2.7
How do you triage snakebite?	Emergency	160	86.0
	Urgent	24	12.9
	Don’t know	2	1.1
Does your health facility have what it takes to manage snakebites?	Yes	90	48.4
	No	91	48.9
	I don’t know	5	2.7
Reasons for which your health facility unable to manage snakebites (n = 89)	Lack or inadequate Anti Snake venom	77	86.5
	Lack of other logistics	10	11.2
	Inadequately qualified staff	2	2.2
Does your hospital have a snakebite management protocol?	Yes	137	73.7
	No	34	18.3
	I don’t know	15	8.1
How confident are you about snakebite management (n = 181)	Not confident	11	6.1
	Fairly confident	79	43.6
	Confident	78	43.1
	Very confident	13	7.2

### Healthcare professionals and their involvement in snakebite management

**Fig 2:**
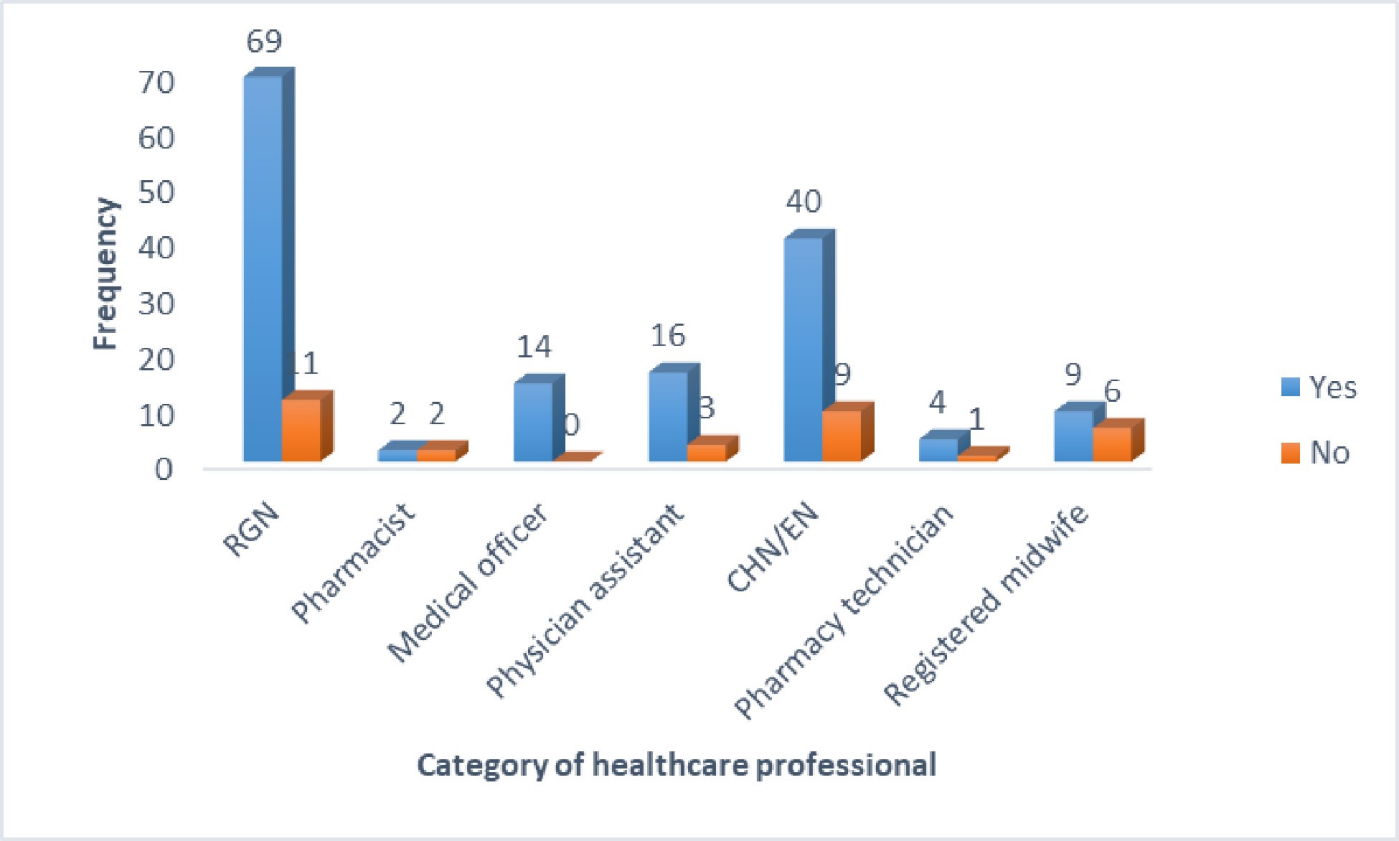
Categories of healthcare professionals and their involvement in the management of snakebite envenoming.

All the medical doctors (100.0%) in this study had ever been involved in snakebite management followed by the registered general nurses (86.3%), physician assistants (84.2%), community health nurses/enrolled nurses (81.6%) 0.0%), and the pharmacy technicians (80.0%). The category of healthcare professionals least involved in snakebite management in this study was the registered midwives (60%) and the pharmacists (50.0%). Fig 2 shows the level of involvement of the health professionals in snakebite management at their health facilities.

### Knowledge of respondents on snakebite management

Table 3 shows the level of knowledge of respondents on snakebite management. The top five best answered questions on snakebite management were; Antivenoms being the only specific antidotes in the management of snake bites by venomous snakes [0.92±0.273 (92.0%)], injecting ASV intramuscularly is as not as effective as using the intravenous route [0.81±0.39 (81.2%)], the 20 minutes whole blood count test (20MWBCT) being the first recommended test for a suspected snakebite envenoming [0.73±0.447 (73.0%)], stating correctly three adverse reactions a patient given anti-snake venom (ASV) may experience [2.01±1.24 (67.0%)] and antivenoms need not be given to all persons suspected of snakebite [0.67±0.47 (67.0%)]. The bottom five areas of least knowledge about snakebite management by the respondents were; ASV being useful for months and years after the labelled expiry date [0.12±0.32 (12.0%)], the intramuscular route being the most appropriate for administering first choice drug used for managing adverse reaction caused by ASV [0.19±0.40 (19.4%)], Adrenaline being the first choice in the management of adverse reactions caused by ASV rather than hydrocortisone which majority, 96 (51.6%) wrongly indicated [0.22±0.42 (22.0%)], a snake bite patient reporting to a facility with a tourniquet applied to the affected limb must be told it is not appropriate, but informed that the tourniquet will not be removed until anti-snake venom is injected [0.22±0.42 (22.0%)] and correctly stating any important biochemical test required in snakebite management [0.31±0.464 (31.2%)]. The overall knowledge score of the respondents on snakebite management was 10.60±5.62 over 22 which is equivalent to 48.2%.

**Table 3:** Knowledge of respondents on snakebite management.

Question	Sub-group/Correctness	Responses		Mean knowledge score (Percentage)
		Frequency	Percentage	
Which of the following will be your comment if a snakebite patient report to your facility with a tourniquet applied to the affected limb?	Not sure of what I will tell the person	17	9.1	0.22±0.42 (22.0%)
	It doesn't matter if it remains or removed since it had at least prevented the movement of the venom	17	9.1	
	It is appropriate, let it remain as we begin treatment	26	14.0	
	It is inappropriate, so remove it immediately	84	45.2	
	****It is not appropriate, but we will not remove it until we have given the anti-snake venom****	42	22.6	
State four recommended first aid procedures to be applied in the right order when you are the first to come to the aid of a person bitten by a suspected venomous snake?^a^	0/4	88	47.3	1.30±1.49 (33.0%)
	1/4	26	14.0	
	2/4	28	15.1	
	3/4	17	9.1	
	4/4	27	14.5	
Which test will you first recommend to determine if a suspected snakebite victim had an injection of venom by the snake?	****20 minutes whole blood count test (20MWBCT)****	135	72.6	0.73±0.447 (73.0%)
	Full blood count	42	22.6	
	Grouping and cross-matching	4	2.2	
	Urinalysis for myoglobinuria	5	2.7	
State any important biochemical test required in snakebite management.^b^	Incorrect	128	68.8	0.31±0.464 (31.2%)
	Correct	58	31.2	
Antivenoms are the only specific antidotes in the management of snake bites by venomous snakes.	No	15	8.1	0.92±0.273 (92.0%)
	****Yes****	171	91.9	
Antivenoms made anywhere in the world is appropriate for all countries.	Yes	108	58.1	0.42±0.50 (42.0%)
	****No****	78	41.9	
Antivenoms should be given to all patients bitten by snakes?	Yes	62	33.3	0.67±0.47 (67.0%)
	****No****	124	66.7	
State 3 indications for the use of antivenom in snake bite.^c^	0/3	44	23.7	1.67±1.184 (56.0%)
	1/3	39	21.0	
	2/3	38	20.4	
	3/3	65	34.9	
State three adverse reactions a patient given anti-snake venom (ASV) may experience.^d^	0/3	40	21.5	2.01±1.24 (67.0%)
	1/3	20	10.8	
	2/3	24	12.9	
	3/3	102	54.8	
Which drug is the first choice in the management of adverse reactions caused by ASV?	****Adrenaline****	41	22.0	0.22±0.42 (22.0%)
	Promethazine	2	1.1	
	Antihistamine	4	2.2	
	Don’t know	40	21.5	
	Hydrocortisone	96	51.6	
	Others	3	1.6	
Which route is the most appropriate for administering the first choice drug used for managing adverse reactions caused by ASV?	Intravenous	122	65.6	0.19±0.40 (19.4%)
	****Intramuscular****	36	19.4	
	Subcutaneous	4	2.2	
	I don’t know	18	9.7	
	Others	6	3.2	
Injecting ASV intramuscularly is as effective as using the intravenous route.	Yes	11		0.81±0.39 (81.2%)
	****No****	151		
	I don’t know	24		
ASV remains useful for months or even years after stated expiry dates.	****Yes****	22	11.8	0.12±0.32 (12.0%)
	No	164	88.2	
In the use of ASV, it is better to give low doses repeated over several days than give high initial doses.	Yes	101	54.3	0.46±0.50 (46.0%)
	****No****	85	45.7	
How long should a suspected snakebite victim who shows no sign of envenoming is detained for observation?^e^	Incorrect	82	44.1	0.56±0.51 (55.9%)
	Correct	104	55.9	
Overall average knowledge score				10.60±5.62/22 (48.2%)

a Recommended first-aid: Move the victim from the area, reassure the victim, remove any constricting materials, and immobilize the whole patient especially the affected limb using a splint or sling.

b Other biochemical tests: plasma creatinine, urea/blood urea nitrogen, and potassium concentrations, elevated aminotransferases, and muscle enzymes (creatine kinase, aldolase, etc.) or hyponatraemia.

c Antivenom treatment is indicated: if/when patients with proven/suspected snakebite develop one or more of the following signs—Systemic envenoming: haemostatic abnormalities such as spontaneous systemic bleeding, coagulopathy or thrombocytopenia; neurotoxicity (bilateral ptosis, external ophthalmoplegia, paralysis, etc.); cardiovascular abnormalities (hypotension, shock, cardiac arrhythmia, abnormal ECG); Acute kidney injury (oliguria/anuria, rising blood creatinine/urea); haemoglobin-myoglobinuria (dark brown/black urine, positive urine dipsticks).

d Headache, nausea, vomiting, urticaria, pruritus, fever, chills, bronchospasm, tachycardia, hypotension, angioedema, abdominal cramps.

e 24 hours. NB. In the table, correct answers were those in bold fonts.

### Association between socio-demographic characteristics and knowledge on snakebite management

Table 4 shows the association between socio-demographic characteristics and knowledge on snakebite management. Male respondents were significantly more knowledgeable about snakebite management than females (11.53±5.67 vs 9.64±5.46; p = 0.022) so also were those who had some previous training on snakebite management than those who were not provided any other form of in-service training (14.14±5.90 vs 9.04±4.75; p <0.001). Previous experience on snakebite management provides significantly better knowledge on snakebite management than one who had never been involved in the management of snakebite (5.17±2.47 vs 3.15±2.38; p <0.001). Respondents working at CHPS zones scored best (14.47±5.48) followed by those at health centres (12.72±6.88) with those at hospitals being the least knowledgeable (9.84±5.18) on snakebite management with the differences in knowledge being statistically significant (p <0.001). There were significant differences in knowledge among respondents based on their district of practice (p = 0.003) with those from the North Tongu District scoring the highest (11.94±5.95), closely followed by those in the Central Tongu District (11.84±5.93) whereas the South Tongu district respondents scored the least (9.84±5.18). There was a significant difference in knowledge on snakebite among the various categories of healthcare professionals (p = 0.031) with the medical doctors obtaining the best mean score of 13.71±6.50 followed by the Pharmacy technician (13.60±6.07) and the pharmacist (13.50±7.77) but the registered general nurses were the worst performers (9.11±4.63). Further grouping of the various categories of health workers based on their core duties found the prescribers being the most significantly knowledgeable group (13.56±6.41; p = 0.017) with the nursing and midwifery group scoring the least (9.98±5.31).

**Table 4:** Association between socio-demographic characteristics and actual knowledge scores on snakebite management.

Characteristic	Sub-group	Mean Score+SD	P-value
Sex	Male	11.53±5.67	0.022*
	Female	9.64±5.46	
Level of health facility	CHPS zone	14.47±5.48	<0.001*
	Health centre	12.72±6.88	
	Hospital	9.84±5.18	
District of health facility	South Tongu	9.13±4.93	0.003*
	Central Tongu	11.84±5.93	
	North Tongu	11.94±5.95	
Area of profession	RGN	9.11±4.63	0.031*
	Pharmacist	13.50±7.77	
	Medical officer	13.71±6.50	
	Physician assistant	11.63±6.08	
	CHN/EN	10.86±5.63	
	Pharmacy technician	13.60±6.07	
	Registered midwife	11.73±6.95	
Professional group	Nursing and midwifery group	9.98±5.31	0.017*
	Prescribers	13.56±6.41	
	Pharmacy group	12.52±6.25	
Training	No training	9.04±4.75	<0.001*
	Received training	14.14±5.90	
Number of years of practice	<5 years	10.80±5.87	0.716
	5–10 years	10.24±5.28	
	>10 years	12.50±6.36	
Ever managed snakebite?	No	3.15±2.38	<0.001*
	Yes	5.17±2.47	

### Comparison between the perceived and actual knowledge scores of respondents on snakebite management and

Table 5 shows the comparison between the perceived and actual knowledge scores of respondents on snakebite management against their sociodemographic characteristics. There was a significant difference between the mean actual and perceived knowledge scores on snakebite management for both males (p < 0.001) and females (p < 0.001) respondents but the females had greater exaggerated confidence than the males (24.9% vs 24.1%). For both the male (p = 0.008) and the female (p = 0.003) respondents, there was a small but significant positive correlation (≈ 0.3) between their perceived and actual knowledge scores. All the age categories had exaggerated snakebite management knowledge scores > 20.0% but those above 39 years had the highest score difference of 43.5%. However, it was only age groups 20 to 29 and 30 to 39 that had the differences between their perceived and actual knowledge scores being statistically significant (p < 0.001). Again, although all age categories had a weak positive correlation between the mean perceived and actual scores, it was only those between 30 and 39 that had a significant correlation (p < 0.001). Whereas health workers in the lowest level of Ghana's healthcare system, the CHPS zones significantly underestimated their knowledge on snakebite management (-25.4%; p = 0.005), their colleagues in the hospitals significantly overestimated their knowledge (37.4%; = < 0.001). It was only respondents in the hospitals that had a moderate but significant correlation between their actual and perceived knowledge on snakebite management. Health workers in the South Tongu District had the highest significant exaggeration of knowledge on snakebite management (46.6%; p < 0.001) as well as a significant and strong correlation between perceived and actual knowledge scores (r = 0.5; p < 0.001). All the categories of the number of years of practice had an exaggerated score but those practicing between 5 and 10 years recorded the highest significant difference score (38.4%; p < 0.001) while those who had worked for less than a year had the lowest significant difference score (17.8%; p = 0.001). Again, whereas those who had practiced for less than 5 years had a moderate but significant correlation (r = 0.3; p = 0.01) between the perceived and actual mean knowledge score, those who had worked between 5 and 10 years had a strong and significant correlation (r = 0.5; p < 0.001). There was over-exaggeration of knowledge on snakebite management whether respondents had over had any form of on-the-job training or not (7.5% vs 38.1%) but those who had no training had a significant exaggeration (p < 0.001) and also a moderate but significant correlation between their actual and perceived mean snakebite management scores (r = 0.4; p < 0.001). Only the pharmacists (-2.3%), midwives (-12.9%), and the pharmacy technician (-22.2%) underestimated their knowledge on snakebite management. Other healthcare workers such as medical doctors (21.5%), physician/medical assistants (24.4%), and registered general nurses (53.7%) exaggerated their knowledge by more than 20.0% but it was only the registered general nurses (p < 0.001) and the physician/medical assistants (p = 0.032) that the differences between their perceived and actual knowledge scores were significantly different. Whereas, there was a moderate but significant correlation between perceived and actual knowledge scores of the registered general nurses (r = 0.4; p < 0.001), that of the physician/medical assistants was a strongly significant correlation (r = 0.5; p = 0.037). Regrouping of the health workers categories based on their core functions found significant differences between perceived and actual knowledge score for the nursing/midwifery group (29.6%; p < 0.001) and the prescribers (23%; p = 0.005) as well as a significant and moderate correlation (r = 0.3; p = 0.002) for the nursing/midwifery group but a significant and strong correction for the prescribers (r = 0.5; 0.005). Both respondents who had in-service training and those without exaggerated their knowledge (26.4% vs 18.9%) but it was only those who had been trained who shows a significant difference between their perceived and actual knowledge scores. Whereas the correlation between the scores for those without training was strong and significant (r = 0.5; p = 0.011), those who had training was significant but weak (r = 0.2; p = 0.042). All the forms of verbal declaration of confidence in snakebite management showed exaggeration of knowledge with those who stated they had no confidence scoring the least difference (6.6%) which was not significant and those who stated they were very confident recording a significantly large difference between actual and perceived knowledge scores (52.7%; p < 0.001).

**Table 5:** Comparing means of respondents perceived and actual knowledge of snakebite management according to their sociodemographic characteristics.

Variable	Subgroup	Frequency	Paired sample statistics		Paired samples test		Paired samples correlation	
			Mean NPS±SD	Mean AKS±SD	Difference (%)	p-value	r	P-value
Sex	Male	93	6.60±1.62	5.32±2.54	1.28±2.62 (24.1)	<0.001*	0.3	0.008*
	Female	88	5.73±1.82	4.48±2.46	1.25±2.55 (27.9)	<0.001*	0.3	0.003*
Age	20–29	78	5.68±1.49	4.52±2.36	1.16±2.60 (25.7)	<0.001*	0.1	0.200
	30–39	96	6.49±1.90	5.22±2.63	1.27±2.57 (24.3)	<0.001*	0.4	<0.001*
	>39	7	7.36±1.18	5.13±2.63	2.23±2.57 (43.5)	0.062	0.3	0.550
Level of facility	CHPS	14	5.21±0.70	6.98±2.01	-1.77±1.94 (-25.4)	0.005*	0.3	0.346
	Health Centre	21	5.86±1.35	6.28±3.06	-0.42±3.04 (-6.7)	0.534	0.2	0.298
	Hospital	127	6.24±1.88	4.54±2.40	1.70±2.27 (37.4)	<0.001*	0.5	<0.001*
District	South Tongu	84	6.23±2.03	4.25±2.21	1.98±2.12 (46.6)	<0.001*	0.5	<0.001*
	Central Tongu	44	6.21±1.53	5.47±2.67	0.74±2.80 (13.5)	0.087	0.2	0.197
	North Tongu	53	6.06±1.51	5.51±2.67	0.55±2.78 (10.0)	0.156	0.2	0.137
Number of years of practice (years	<5	109	5.90±1.54	5.01±2.64	0.89±2.71 (17.8)	0.001*	0.3	0.010*
	5–10	69	6.56±2.03	4.74±2.38	1.82±2.30 (38.4)	<0.001*	0.5	<0.001*
	>10	3	7.33±1.53	5.91±2.08	1.42±1.87 (24.0)	0.318	0.5	0.667
Ever had training on Snakebite management?	No	125	5.80±1.77	4.20±2.14	1.60±2.24 (38.1)	<0.001*	0.4	<0.001*
	Yes	56	7.01±1.45	6.52±2.62	0.49±3.09 (7.5)	0.239	-0.1	0.558
Profession	RGN	78	6.47±1.93	4.21±2.08	2.26±2.16 (53.7)	<0.001*	0.4	<0.001*
	Pharmacist	4	6.00±2.94	6.14±3.53	-0.14±2.18 (-2.3)	0.909	0.8	0.213
	Medical doctor	14	7.57±1.01	6.23±2.95	1.34±2.09 (21.5)	0.085	0.4	0.132
	Physician/Medical Assistant	19	6.58±1.47	5.29±2.76	1.29±2.43 (24.4)	0.032*	0.5	0.037*
	CHN/Enrolled nurse	48	5.56±1.38	5.02±2.52	0.54±2.69 (10.8)	0.169	0.2	0.317
	Pharmacy Technician	4	5.75±0.96	7.39±0.68	-1.64±1.27 (-22.2)	0.082	-0.2	0.826
	Midwife	14	4.86±1.99	5.58±3.12	-0.73±2.45 (-12.9)	0.296	0.6	0.018*
Professional group	Nursing/Midwifery	140	6.00±1.79	4.63±2.39	1.37±2.59 (29.6)	<0.001*	0.3	0.002*
	Pharmacy staff	8	5.88±2.03	6.76±2.44	-0.88±1.84 (-13.0)	0.215	0.7	0.065
	Prescribers	33	7.00±1.37	5.69±2.84	1.31±2.50 (23.0)	0.005*	0.5	0.005*
Ever managed snakebite?	No	29	3.97±1.57	3.34±2.42	0.63±2.19 (18.9)	0.134	0.5	0.011*
	Yes	152	6.61±1.47	5.23±2.44	1.38±2.63 (26.4)	<0.001*	0.2	0.042*
Level of confidence in managing snakebite	Not confident	11	2.91±1.76	2.73±2.36	0.18±2.14 (6.6)	0.784	0.5	0.124
	Fairly confident	79	5.21±1.08	4.34±2.35	0.87±2.41 (20.0)	0.002*	0.2	0.126
	Confident	78	7.17±1.00	5.66±2.58	1.51±2.78 (26.7)	<0.001*	-0.01	0.907
	Very confident	13	8.81±0.69	5.77±1.39	3.04±1.65 (52.7)	<0.001*	-0.2	0.630

NPS = Nominal perceived score.; AKS = Actual Knowledge Score; SD = Standard deviation * Statistically significant

## Discussion

The outcome of a disease condition depends on several factors including the human beings involved in the process; an assertion supported by the Institute of Medicines’ definition of health care quality as the degree to which health care services for individuals and populations increase the likelihood of desired outcomes and are consistent with current professional knowledge [10]. Snakebites have become an event that claims the lives of between 81,000 and 138,000 persons annually most of whom are poor persons in developing countries involved in agriculture to produce food for their nations and for export to bring foreign exchange to their countries [4]. Although some victims of snake bites seek the services of traditional healers many others seek medical assistance from orthodox health facilities where the provision of quality healthcare service can ensure the survival of a snakebite victim or eliminate or reduce any post-exposure morbidity [6,11].

Increased productivity had been reported among professionals that are confident about the work they do, which ultimately increases the gratification they derive from the job [12]. Lack of confidence by a healthcare professional can result in feelings of inadequacy, frustration as well as helplessness which can result in increased medical errors which thereby increases the chance of health worker related deformity or death occurring [13,14]. As much as confidence is needed in the performance of duty, the over-exaggeration of one's ability is also detrimental. Since envenomation after a venomous snakebite can quickly affect various body systems and ultimately leading to death, if the management of the victims and possible adverse effects of the anti-snake venoms are not executed well by a highly knowledgeable and skills health worker, the prognosis may not be good enough. This study found over-exaggeration of knowledge on snakebite management across various sociodemographic classifications when the actual knowledge scores on snakebite management were compared with their presumed level of knowledge before the completion of the knowledge assessment section of the questionnaire. The overall knowledge of health workers on snakebite management in this study was below average [10.60±5.62/22 (48.2%)]. This poor knowledge of health care professionals on snakebite management seems to be the same irrespective of the level of development of the health sector of countries. Studies from the United Kingdom and Hong Kong recorded low knowledge on snakebite management which is the same in several developing countries such as Laos PDR, Bangladesh, Cameroon, Nigeria [15–20]. Males in this study were significantly more knowledgeable about snakebite management than their female counterparts who even significantly exaggerated their knowledge level. The lower knowledge base of females on snakebite management can be attributed to the fear women generally have for snakes [21]. Michael, et al., (2018) did not, however, find any association between the sex of respondents and their knowledge level [20]. It is not clear why the females significantly exaggerated their knowledge which is in contrast with the results of studies that found men to exaggerate their capabilities and was also less honest [22,23]. In this study, health workers in the hospitals were significantly less knowledgeable about snakebite management that colleagues in the lowest level health facility in Ghana, the CHPS zone (p < 0.001) who as well exhibited overestimated confidence of 37.4% compared to the under exaggeration of -25.4% by those working in the CHPS zones. For health workers in the hospitals, there seems to be a moderate but significant correlation between the perceived and actual knowledge on snakebite management (r = 0.5; p < 0.001). The disparity in knowledge levels by the higher and lower-level health facility can be due to the more exposed those in CHPS zones are to snakebite issues than those in the hospitals. These results then place snakebite victims that are sent or referred to these higher-level health facilities at a higher risk of mismanagement. Among the various health professions, the medical doctors in this study were significantly the most knowledgeable and the nurses least knowledgeable on snakebite management just as reported in some previous similar studies [17,19]. This is understandable since the medical doctor plays the leading role in the management of all cases in the hospitals. The 21.5% over-exaggeration of knowledge by the physician and a higher and significant (24.4%; p = 0.037) overestimation of knowledge by the physician/medical assistants can be detrimental to their effective management of cases. The nurses who exhibited the least knowledge level just as in some earlier studies were also the same health professional group that overestimated their knowledge level the most (53.7%, p < 0.001) [17–19]. This study found those who had ever managed or ever been trained on snakebite management to be significantly more knowledgeable than those who never managed a case or had no previous training (p < 0.001). The effect of training or experience on better management of snakebite had also been observed in some earlier studies in Cameroon, Lao PDR, and Nigeria [17,19,20]. Health professionals who had no training but mostly obtained their skills by observing their senior colleagues rather overestimated their knowledge level (38.1% vs 7.5%). On the other hand, respondents who had ever managed cases although significantly more knowledgeable (p < 0.001), also overestimated their knowledge level (26.4% vs 18.9%). This over-exaggeration of snake management skills for the untrained and even those who had ever managed snakebite cases can adversely affect the management of snakebite victims as they will be inappropriately more confident as they even administer or manage such cases wrongly. The effect of high confidence level on the knowledge of respondents was succinctly exhibited when differences between perceived and actual knowledge scores were analyzed. The more confidence a health worker expresses, the higher the overestimation of knowledge; those not confident (6.6%) and very confident (52.7%). Although the overall knowledge on snakebite management may be poor, there were some areas where they showed some good knowledge especially those about the 20 minutes whole blood count test, anti-snake venom being the only specific antidote for envenomation and the best route for administering being intravenous. Management of ASV adverse drug reaction (ADR) was rather poorly answered. For more than half (51.6%) of respondents to opt for hydrocortisone rather than adrenaline (22.0%) as their 1^st^ choice in the management of ASV associated adverse drug reaction is a source of worry. This result is even better than a study involving only physicians in a developed country such as Hong Kong, where 57% also opted for hydrocortisone and other antihistamines to manage ASV-induced anaphylactoid reactions [15]. However, up to 90.8% of health workers in the Laos PDR study chose adrenaline as their drug of choice for the management of ASV induced adverse drug reaction [17]. Respondents in this study also exhibited paucity in knowledge on the route of administrating of ASV adverse reaction antidote (19.4%). This poor knowledge of the management of ASV-associated ADR seems to be common among health workers across the world as it was reported in India and Hong Kong [15,24]. ASV-associated ADRs are common and known to occurs between 25% and 62% of victims of snakebite which shows that some morbidity and mortality of snakebites are may not be due to the envenomation only but also mismanagement of the ADR associated with its management [25–29].

Results of this study being the first in Ghana we believe should make managers of health systems in Ghana and other developing countries see the need to include snakebite management in the curriculum of their health training institutions. Again, they will formulate policies that will ensure more frequent in-service training on snakebite management for all health workers. Governments should also stock health facilities in rural areas with anti-snake venoms since that was the most stated limitation most health workers indicated as one that affects their facilities' ability to manage such cases. Almost half of the respondents have managed between 1 and 5 snakebite cases within half a year, which shows that snakebite is a disease in these rural Tongu districts. Notwithstanding the usefulness of this study to public health, it is important to state some important limitations of this study. In the first place, the study took place in only three out of about two hundred and sixty districts of Ghana so may not represent the situation across the country. Again, the generalization of the results may not be appropriate since convenience sampling rather than randomization was used in the selection of the nursing professionals which introduced some biases in the selection of this category of respondents. It was also not possible to know if the target respondents completed the questionnaire by themselves and even whether they used some reference materials in answering some of the questions. The small sample size of the medical doctors and the pharmacy staff is another limitation worth noting since results from this cadre of health care workers cannot adequately represent the true story about their knowledge about snakebite management.

## Conclusion

There is a deficit in knowledge in the management of snakebite cases among health care professionals in the three Tongu districts of the Volta region with a significant number overestimating their knowledge levels which can lead to mismanagement of victims of snakebite. This presents a serious limitation in the effective management of snakebite victims in an area in Ghana whose main occupation is agriculture-related for which snake envenoming is a potential occupational hazard. Managements of Health training institutions would have to consider augmenting their training curriculum to include lessons on snakebite management since these bites resulting from the snake and human conflicts will continue since agriculture continues to be the mainstay of the economies of developing countries such as Ghana. Results from this study would awaken health policymakers to appreciate the need for more in-service training of health professionals on snakebite management as well as issues related to the management of adverse drug reactions associated with anti-snake venoms. The nursing group who are the most populous of all health workers and are responsible for the healthcare needs of many persons in rural areas of Ghana exhibited the least knowledge on snakebite management, more training activities must be directed at them so safe the lives of the many poor persons who through their agricultural activities continue to provide food for the many living in urban areas.
