# Supplementary material for: Health professionals' overestimation of knowledge on snakebite management, a threat to the survival of snakebite victims—A cross-sectional study in Ghana
Source: PLoS Negl Trop Dis. 2021 Jan 19;15(1):e0008756. doi: 10.1371/journal.pntd.0008756 (PMC7846110; doi:10.1371/journal.pntd.0008756)
Supplement: Attachment — Submitted filename: Letter - Responses to reviewers comments.docx [file pntd.0008756.s001.docx]

Dear Editor

We have considered the comments of the reviewers and have given our responses accordingly. We disagreed with some of the comments and we gave reasons for the disagreement for your consideration. We have also agreed with some comments and have subsequently corrected these errors. We appreciate the comments of the reviewers and we believe it had improved the quality of the manuscript. We have also used Grammarly.com to detect and correct some errors. Thanks very much.

Evans Ameade

Reviewer 1

**COMMENT 1**

1. The authors' hypothesis (50% of people showing sufficient knowledge of the management of snakebite envenoming) is speculative and unfounded.

**RESPONSE 1**

In using Cochran formula to calculate sample size, the p (the estimated proportion of an attribute that is present in the population) can be based a previous study. But in this case, I did not find any study which assessed the proportion of various categories of health care professionals on their knowledge on snakebite management. In such a situation, it is acceptable to give *p* the maximum variability which is 50% or 0.5 (Refer to <http://www.gjimt.ac.in/web/wp-content/uploads/2017/10/2_Glenn-D.-Israel_Determining-Sample-Size.pdf> and <http://citeseerx.ist.psu.edu/viewdoc/download?doi=10.1.1.486.8295&rep=rep1&type=pdf> ). The 50% used was therefore in my view not speculative and unfounded.

**COMMENT 2**

2. The objective of the study is not clearly established. The design of the study aims only to establish a state of knowledge of an ill-defined audience.

**RESPONSE 2**

We have now clearly stated the objectives of the study are requested for by reviewer 1. We however are not sure how the reviewer concluded that the respondents were ill-defined. I stated clearly under the study population that the respondents were health providers and listed the categories they belong to. “The study population were health care providers namely; pharmacists, physician/medical assistants, medical doctors, pharmacy technicians as well as midwives and nurses of various categories who work in hospitals, health centres and CHPS compounds in the study area”. Again, snakebite management according to the WHO is an emergency situation so all levels of healthcare and various categories of health care providers are to be trained to effectively manage snakebite envenoming to reduce mortality and morbidity associated with it. (Refer to pp 114 to 115 of the WHO Guidelines on snakebite management from <https://apps.who.int/iris/handle/10665/249547>) which said *“Management of snakebites at different levels of the health service: can contribute to the management of patients with suspected snakebite. In rural areas, where snakebites are most common, transfer to a hospital may not be feasible, and so a lower level of health facility must cope with these medical emergencies. Training of doctors, nurses, dispensers, health assistants and paramedics in diagnosis (including snake identification), early management and indications for and practicalities of administering antivenom is essential”*

**COMMENT 3**

3. The sample size is large but the power is not determined.

**RESPONSE 3**

Sample size and power value are often based on two group comparison. This is not a study that had a controlled group and a test group. Am not sure how the power can be determined for the sample size of this study. Can reviewer 1 provide some information on how to calculate the power from a sample size calculated using the Cochran formula? Using the Cochran formula with the appropriate assumptions as stated in the manuscript should give an acceptable sample size.

**COMMENT 4**

4. There is no statistical analysis to relate participants' responses to their occupational category and their involvement in the management of snakebite envenoming.

**RESPONSE 4**

We have included a bar chart (Figure 1) showing the occupational category and their involvement in management of snakebite envenoming. Hope it will satisfy the concern in comment 4.

**COMMENT 5**

5. There is no analysis plan and justification presented explaining the structure and details of the questionnaire.

**RESPONSE 5**

We have the analysis plan is under ‘Statistical analysis’ part of the methodology. The structure of the questionnaire is under ‘ Data collection instrument and technique’ with even the information provided under ‘Data measurement’ detailing how the scoring of the questions were done. Will be glad if the reviewer 1 can help us better understand his or her comment if our response did not satisfy the requirement indicated by comment 5.

**COMMENT 6**

6. The results are clear but presented in an incomplete way.

**RESPONSE 6**

Will be glad if reviewer 1 will let us know which other additional information that may have to be added to make results complete. We were also guided by the fact that there is a limit to the number of tables or figures one can include in manuscripts. We have 5 result tables in this manuscript. The study sought to determine how knowledgeable health workers were on snakebite management and whether their perceived knowledge was not an overestimation when compared to the actual knowledge level which was assessed using various questions. So, Table 3 measured their knowledge on snakebite management. Table 4 sought to determine if there were associations between their knowledge and some socio-demographic characteristics of the respondents. Table 5 then compared their perceived knowledge level which was gotten from their response to a question which require they indicate between 1 and 10 how they will assess their own level of knowledge on snakebite management. Table 5 further showed if there were statistically significant differences between their perceived and actual knowledge levels based on some characteristics including the health professional group they belong to. We have meanwhile included in the manuscript a figure showing the occupational category and their involvement in snakebite envenoming. Hope that should complete the results.

**COMMENT 7**

7. The figures (tables, images) are of sufficient quality.

**RESPONSE 7**

Well noted.

**COMMENT 8**

8. The conclusions are trivial and limited to the objectives of the study, i.e. to show no more than insufficient knowledge of health workers about snakebite envenoming.

**RESPONSE 8**

Well noted. I have therefore expanded that conclusion section

**COMMENT 9**

9. The limitations of the study and analysis are not described. The selection of participants is based on respondents to an open online survey, without control over selection by designers. Furthermore, the analysis did not establish a link between participants' responses and their status or responsibility in the management of snakebite envenoming.

**RESPONSE 9**

Please refer to the last paragraph under the discussion section where the limitations were indicated. We have however added some more limitations as suggested by reviewer 1. On the analysis linking participant’s response and their status or responsibility in the management of snakebite envenoming, we believe inclusion of figure 1 will take of that concern.

**COMMENT 10**

10. The authors do not discuss how the data can be useful in advancing our understanding of the topic in question.

**RESPONSE 10**

We have included statements in the conclusion section of the manuscript on how the study can help to bring better appreciation of the topic under discussion and hope that answers the concern expressed by reviewer 1.

**COMMENT 11**

11. The relevance of public health is taken into account without comments, suggestions or recommendations for intervention based on the results.

**RESPONSE 11**

The additional statements in the conclusion brings up some recommendations based on the results

**COMMENT 12**

12. The manuscript "Health professionals’ over estimation of knowledge on snakebite management, a threat to survival of snake bite victims - A cross-sectional study in Ghana" by Ameade et al. has already been submitted to PLoS NTD and recently rejected. Although the topic is very important and the authors have extensively revised the article, there are still significant flaws in the methodology, and interpretation and capitalization of the results. One of my main concerns is the choice of sets of questions between the first manuscript and the second. Essential questions disclosed in the second manuscript (tables 2 and 3) did not appear in the first and, conversely, important questions presented in the first manuscript (table 4: Knowledge of snake bite, Q21 to Q35) are not included in the second… More generally, none of the answers to the questions concerning Knowledge on management of snake bites, Q36 to Q60 appeared in the first manuscript.

**RESPONSE 12**

The manuscript rejected by Plos NTD earlier was different from this one although data was from the same project. The first manuscript was on just respondent’s knowledge about snakes and snakebites. This manuscript was mainly for assessing health workers knowledge on snakebite management hence you will not find the Q36 to 60 in this second manuscript as well as some other questions which were not relevant to this new topic. The data collected was found to be too huge for one publication hence the decision by the authors to break this project into two manuscripts.

**COMMENT 13**

13. Another major concern is the ambiguity of the objective sought by the authors. The subject is essential but its importance is linked more to the knowledge necessary for health personnel to plan their training and perform their tasks, than to the level of current knowledge. Knowing the latter is only of interest if training is carried out and a second survey assesses the acquisition of knowledge and the relevance of the training. In addition, knowledge analysis should be correlated to the category of personnel and be used as an argument for proposing appropriate solutions.

RESPONSES

Will be glad to understand the ambiguity indicated by reviewer 1. This manuscript we think is clearly looking at what healthcare professionals who because of their location in a rural environment are involved in the management of snakebite envenoming. The health system in Ghana is such that in most rural settings, the CHPS (Community Health Planning and Services) /facilities are the first point of call when a person becomes unwell. These health facilities are manned mainly by registered general nurses or community health nurses or another category of nursing group called the enrolled nurses. Personnel in these CHPS facilities are therefore called upon to manage even snakebites without the supervision of a medical officer that may be several tens of kilometres away. You will notice in figure 1 that after the doctors, the registered general nurses were the healthcare professioinal that had were heavily involved in the management of snakebite envenoming even more than the physician assistant who may be found in health centres which are higher-level health facilities than the CHPS compounds. These nurses who may not have been trained specifically in emergency medicine are managing snakebite incidents anyway hence the need for this study to ascertain if they really have the requisite knowledge necessary to manage this emergency. If we understand reviewer 1’s statement “Knowing the latter is only of interest if training is carried out and a second survey assesses the acquisition of knowledge and the relevance of the training”, I must say that this was a cross-sectional study and not a longitudinal study which will require a second survey to ascertain effect of training on their service delivery. In any case, a question was asked to know those who had obtained any post-qualification training which was then analyzed together with their knowledge scores (Table 4) with the results showing that previous training is significantly associated with a person’s knowledge on snakebite management. Reviewer 1 also asked that knowledge analysis should be correlated with the category of health personnel. We think Tables 4 and 5 show knowledge levels based on personnel category and also which category’s perceived knowledge was significantly different from the actuals. This was also well explained under the discussion section.

**COMMENT 14**

14. Finally, I do not trust surveys launched on the Internet without a clearly defined strategy, not targeting an accurate audience and without specific objectives for suitable exploitation of the results.

**RESPONSE 14**

Internet based surveys with their limitations also come with several advantages. (Refer to <https://explorance.com/blog/10-key-advantages-of-using-online-surveys-2-2/#:~:text=Convenience%3A,increase%20engagement%20and%20response%20rates>. & <https://www.cfrinc.net/cfrblog/online-surveys-advantages-disadvantages>) Not sure what reviewer 1 meant by ‘without a clearly defined strategy’. The strategy was to get all physicians, pharmacists, pharmacy technicians as well as physician assistants in selected heath facilities (including all the hospitals) in the Tongu districts surveyed. Great efforts were made to get all of them involved. The response rate for the physicians was 82.0%, pharmacists (80%), Pharmacy technicians (83%) and the physician assistant (73%) which is good enough. The response would probably be worse if it had been paper-based questionnaire. Face to face interview would also be problematic since earlier efforts to meet some of them physically during the piloting of the study tool did not materialize. In our recommendation under conclusion, it was clearly stated that the results of this study brings to the fore the need for inclusion of snakebite management into the curriculum of health training institutions and also provision of in-service training after health workers qualifying to practice.

**COMMENT 15**

15.    However, adding these answers and making corrections to the first manuscript does not change most of the criticisms made by reviewers on the first submission.

•    The questionnaire has not been previously validated, in particular by snakebite envenoming relevant professionals.

**RESPONSE 15**

In the first place, this manuscript is not exactly same as the first one beside involving same respondents. If there are some questions to be answered and some corrections made, I believe it is the peer review process that will make it better. We disagreed with a good number of comments made mainly by one of the reviewers of the first manuscript but since the paper was rejected, there was no opportunity to explain some of the concerns of the reviewers and debunk some of the assertions that were in error. Secondly, it was stated clearly under methodology that the questionnaire is a de novo one. We did not find those questionnaires used in the earlier studies comprehensive enough hence the need to develop a completely new one. The researchers thoroughly discussed the questions and their answers to ensure face validity of the study tool. The reliability of the study tool was further assessed after analyzing the results of the piloting exercise which gave a good Cronbach alpha value of 70% or 0.7 (indicated under data collection instrument and technique). The study tool was therefore considered by the researchers as appropriate for the study.

**COMMENT 16**

•    The calculation of the number of participants required is not very interesting compared to the selection of participants, which should be more rigorous and better documented to confirm that they are representative of the health personnel.

**RESPONSE 16**

Not sure what reviewer means by calculation of number of participants not being interesting. In calculating sample sizes using the Cochran formula is one of the many acceptable ways and therefore not sure what reviewer means by ‘calculation of the number of participants required not very interesting’ For the category of health workers that had small numbers, census sampling was applied. For nursing category, convenience sampling seems to the only plausible and cost-effective method. We were dealing with health care professionals who will all not be at post at the same time and also, we cannot bring all of them together under one roof so that random sampling could be applied. For the nursing cadre of health workers, most of them work on shift basis such that while other will be found during the day, some other work at night. Using the online questionnaire seem to be the best option available to the team to undertake this research.

**COMMENT 17**

•    The rationale for the choice of the sample (involving a priori self-responders) is not clear, nor that of the selected centers, especially in relation to their regular management of snakebites.

**RESPONSE 17**

As we indicated earlier, in Ghana’s health system, the CHPS facilities are manned by non-medics and they are the first point of call in rural settings. It is therefore important to include the nursing and the midwifery brigade into the study. The pharmacy workers are involved in advising the medical staff on drugs and are responsible for the stocking of these important medications in the management of snakebite envenoming hence are also critical workforce to part of this study. The figure 1 shows that it was not only medics who are managing snakebite cases in the study area hence it was prudent to include all other healthcare professionals in this study. Meanwhile, the WHO’s policy on snakebite management requires that all categories or health workers must know how to manage snakebites (Refer to pages 114 to 115 of the WHO document on snakebite management from <https://apps.who.int/iris/handle/10665/249547>). It must also be noted that in well-developed health systems, envenomings are best managed by critical care/emergency specialist but in the case of developing nations such as Ghana, even house officer and junior medical doctors who may be working at district hospital will be called in to manage snakebites. The study again showed that regular snakebite management is being done at all the levels of healthcare including the CHPS facilities.

**COMMENT 18**

•    The age of the respondents is less than 40 years for more than 95% of the participants, which is probably not representative of the health personnel in this region of Ghana. In addition, the participant sample lacks of more experienced people who could play a crucial role in the training of the youngest.

**RESPONSE 18**

It is a valid observation but it is not surprising because in many rural settings, health professionals who may accept to work or compelled to work there will be the young ones with the older ones who may be specialist or consultants working in regionals as well as teaching hospitals in the cities. In this study, 14 out of the 17 medical doctors in the three districts were sampled with the oldest being 52 years (not accurate that experienced ones were not involved in the study) and an average age of 33 years. For the pharmacy group, the oldest was 40 years with an average age of 34. That is the really in our health systems and this data is the actual representation of health workers in the Tongu districts of Ghana. One cannot discount the fact that among the nursing or midwifery group, there may be some older ones but majority will be those below 30 years.

**COMMENT 19**

•    The distribution of responses according to categories of health personnel is not analyzed. This is all the more regrettable as one cannot expect the same level of knowledge from all categories of health workers: more knowledge is expected from a doctor than from a nurse.

**RESPONSE 19**

The distribution of responses according to categories of health personnel can be found in Tables 1 and 5. It is true that the medical doctor is expected to be more knowledgeable on this subject than the nurse but depending on the area of work of the doctor, you may have a nurse who is a lot more exposed to snake envenoming management and had gotten more in-service training to be more knowledgeable than a doctor. However, this study found a significant difference between the categories of health care professionals which confirms the doctor as the most knowledgeable in snakebite management although their score was not too far from that of the pharmacist or pharmacy technician. (Table 4). The WHO however requires that training of doctors, nurses, dispensers, health assistants and paramedic in diagnosis (including snake identification), early management and indications for and practicalities of administering antivenom is essential (Refer to pp 114 to 115 of the WHO Guidelines on snakebite management from <https://apps.who.int/iris/handle/10665/249547>)

**COMMENT 20**

•    Some relevant questions are missing such as, for example, the training received after school by the participants (Q2, table 2).

**RESPONSE 20**

Is it the kind of post-qualification training reviewer wishes to know? The question as presented in the questionnaire was “Since you started practising as a health care professional, have you ever received training on snake bite management through a seminar or workshop?” So, we clearly indicated the type of acceptable training programme. Someone may indicate their post-qualification training being ‘on the job’ or self-tuition which we cannot be sure of its quality hence the need to clearly list seminars or workshops as the acceptable programme. We have meanwhile added ‘through seminars or workshops’ to the Q2, in Table 2.

**COMMENT 21**

•    More important, some of the answers suggested by the authors in Table 3 do not seem appropriate:

-The indications for treatment with antivenom (SAV) do not include edema or necrosis, which are possible indications.

**RESPONSE 21**

We have made effort to address the perceived ‘inappropriateness’ of the answers as stated by reviewer 1. We must state that the answers to all questions were from the WHO guidelines for the management of snakebites (Refer: <https://apps.who.int/iris/handle/10665/249547>. and

<https://apps.who.int/iris/bitstream/handle/10665/204458/9789290231684.pdf?sequence=1&isAllowed=y>)

The indications for the use of SAV was clearly stated under Table 3 and we did not include oedema and necrosis. Will be glad if reviewer 1 can show us where we indicated edema or necrosis. Or is reviewer 1 indicating that edema and necrosis should also be added to the signs as stated as a legend under Table 3? Edema will occur as a result of haemostatic abnormalities and kidney injury caused by the venom.

**COMMENT 22**

- The first choice of drug in the management of adverse reactions caused by SAV depends on the severity of the symptoms. For pruritus, hives, or mild general disorders (malaise, nausea, and fever), adrenaline – and possibly corticosteroids - are certainly not the appropriate first-line treatment. Adrenaline should be used in case of anaphylactic shock and corticosteroids act more slowly than antihistamines, which are very effective and suitable for the treatment of moderate allergy. However, the question does not refer to the level of severity, which is essential in the event of an adverse reaction. In addition, promethazine is an antihistamine: why suggest both?

**RESPONSE 22**

- We did not suggest promethazine and antihistamine for respondents to choose from. The list of the drugs for the management of the adverse drug reaction in Table 3 were stated by the respondents. It was an open-ended question with the respondents being asked to indicate the most appropriate medication. As shown in the table, those who indicated (4 respondents) ‘Antihistamine’ did not specify the type of antihistamine.
- We are not sure the source of the protocol used for the management of adverse reactions caused by SAV being referred to by reviewer 1. We referred to the WHO protocol for the management of adverse reaction caused by SAV, this was what was stated on page 134 subsection 6.7.5.4 of the WHO guideline on snakebite management from <https://apps.who.int/iris/handle/10665/249547> *“Because severe, life-threatening anaphylaxis can evolve so rapidly, epinephrine (adrenaline)should be given at the very first sign of a reaction, even when only a few spots of urticaria have appeared or at the start of itching, tachycardia or restlessness.”* This statement is in variance with reviewer 1 suggestion that adrenaline is only administered when anaphylactic shock occurs. Reading through the WHO guideline have some studies suggesting the administration of adrenaline even before giving any anti-snake venom and highly recommended (Refer to: page 134 subsection 6.7.5.4 of the WHO guideline from <https://apps.who.int/iris/handle/10665/249547>

***Recommendation: based on the results of a powerful and well-designed trial in Sri Lanka, routine use of prophylactic adrenaline is recommended before antivenom treatment, except in those older patients in whom there is evidence or suspicion of underlying cerebrovascular disease and when the particular antivenom in use has a proven low incidence of reactions (<5%). The adult dose of epinephrine (adrenaline) is 0.25ml of 0.1% solution (0.25 mg) by sub-cutaneous injection”***

***(children 0.005 ml/kg body***

***weight of 0.1% solution) (T).***

**COMMENT 23**

- The route of administration of first choice drug in the management of adverse reactions should be deep subcutaneous rather than intramuscular in case of hemorrhagic syndrome, which is common in snakebite envenoming in sub-Saharan Africa.

**RESPONSE 23**

According to the WHO guideline, this was the statement on the route of administration of Adrenaline *“Epinephrine (adrenaline) is given intramuscularly (ideally into the upper lateral thigh) in an initial dose of 0.5 mg for adults, 0.01 mg/kg body weight for children.”* (Refer to page 134 subsection 6.7.5.4 of the WHO guideline ( <https://apps.who.int/iris/handle/10665/249547> )

Even the WHO guideline for management of snakebite in Africa did not clearly indicate the IM route not SC (Refer to page 81 of that document: <https://apps.who.int/iris/bitstream/handle/10665/204458/9789290231684.pdf?sequence=1&isAllowed=y>

**COMMENT 24**

- It does not seem wise or appropriate to suggest administration of SAV "months or even years" after expiration date. The effectiveness is probably retained long after the expiration date, but safety is not proven. However, a scientific journal cannot suggest violating a legal provision...

**RESPONSE 24**

In a resource constraint environment such as rural Ghana, it is important that healthcare professionals should be in the know on the effectiveness of SAV even when the date of expiry elapses. WHO documents are internationally accepted source of reference hence we do not with all humility agree with reviewer 1 on this score. It is not a violation of any legal provision if the WHO can state it clearly in its guideline to all health workers. According to the WHO, “*Ideally, antivenoms should be used before the stated expiry dates but, provided that they have been properly stored, they can be expected to retain useful activity for months or even years after these dates (WHO, 1981; O’Leary et al., 2009)*. TRC (Thailand Red Cross) antivenoms retain their potency after 5years even when stored temperatures of 25-50 °C (Refer to page 136 subsection 6.7.6 of the WHO guideline found at <https://apps.who.int/iris/handle/10665/249547> )

**COMMENT 25**

- The period a suspected snakebite victim to be detained, also depends on the time between the bite and presentation to the hospital. In the absence of any clinical sign, 24 hours seems very long and expensive for the patient.

**RESPONSE 25**

The 24 hours observation ais also a recommendation by the WHO for the management of snakebite in Africa (Read: <https://apps.who.int/iris/bitstream/handle/10665/204458/9789290231684.pdf?sequence=1&isAllowed=y> page 59, subsection 9.3 and it reads *“Patients bitten by snakes should ideally be observed in hospital for at least 24 hours after the bite”*

**COMMENT 26**

- At least one of the questions concerns the events between January and June, while participants started to answer in May (lines 122 and 123), which is not consistent.

**RESPONSE 26**

The piloting of the study tool started in May but the actual data collection was in June, hence the question which asked to know the number of snakebite cases treated half year from January, to June, 2019

Reviewer 2

Thanks for the opportunity of reading this very interesting paper. It is a very important body of evidence, which could rapidly translate into action.

**COMMENT 1**

1. The methodology is transparently explained. However, no random cluster sampling was used, a "convenience sampling" was used, and only 3 out of 61 districts were surveyed (as stated in the limitations).

**RESPONSE 1**

Using random sampling for an online survey is a challenge since those who might have been selected may not be available or willing to complete the form. As you may have observed, for the category of healthcare professionals whose numbers are on the low side, census sampling was applied. Convenience sampling is therefore the most appropriate method for a study such as this when the respondents are scattered in various health facilities and they are all not available at the same time for any randomization to occur. The other possible randomization will involve getting the names and WhatsApp numbers of each of the workers at the health facility and then probably use Excel to randomize them. We initially wanted to use random sampling for the nursing and midwifery group hence effort was made to obtain such information on the workers from the health facilities but that presented a huge challenge since the managers of these health facilities did not have it or were unwilling to give that out. There are 18 administrative districts in the Volta region of Ghana and it was out of the number that the 3 neighbouring Tongu districts were selected. The vegetation across the reasons is not uniform and the intention of the authors was to look at the situation in an area having the savanna type of vegetation which supports the survival of many species of snakes.

**COMMENT 2**

2. The main results (knowledge deficit) are however sufficiently evident.

**RESPONSE 2**

Well noted. Thanks

**COMMENT 3**

3. Sample size calculation shows that the power expected was not fully achieved but overall, the sample seems sufficient for the large group conclusions. However, subgroup analyses could be biased by the lack of power (samples too small) to infer conclusions, given the diversity of health facility type/ profession type. Therefore, I would address this more among the study limitations.

**RESPONSE 3**

Sample size and power value are often based on two group comparison. This is not a study that had a controlled group and a test group. Am not sure how the power can be determined for the sample size of this study. Can reviewer 1 assist by providing a reference material? The sample size was gotten using the accepted Cochran formula. We appreciate the challenge of sample size for some categories of the health care professional hence the decision to undertake the study in three districts instead of one of them. We will include this as a limitation under the discussion section.

**COMMENT 4**

4. Limitations. The chapter should be a bit longer.

**RESPONSE 4**

Additional limitations included

**COMMENT 5**

5. Table 5 is slightly too complex for the non-statistician reader (I was trained so I understand but I am not sure that all details, like all means, in this table are necessary).

**RESPONSE 5**

Not sure how it could be simplified. It is the results in this table that brought up the issue of over or under estimation of the knowledge levels of the healthcare professionals on snakebite management.

**COMMENT 6**

6. The conclusions are well supported and clear with the exception of the knowledge on snake species (photos in your google-form) which don't seem very relevant to me.

**RESPONSE 6**

The knowledge on snake section of the google form was not used in this manuscript since the topic for this manuscript was in relation to healthcare professionals and their knowledge levels on snakebite management. However, identification of snakes is very important in the management of snakebites as stated by the WHO. Able to differentiate between venomous and non-venomous snakes will prevent unnecessary administration of antivenoms which by itself can also cause adverse reactions leading to death. (Refer to pp 114 to 115 of the WHO Guidelines on snakebite management from <https://apps.who.int/iris/handle/10665/249547>) which said *“Training of doctors, nurses, dispensers, health assistants and paramedics in diagnosis* ***(including snake identification****), early management and indications for and practicalities of administering antivenom is essential”*

**COMMENT 7**

7. Health staff (nu, mw, md) do not need to know about snake recognition: knowledge should rather be based on CLINICAL MANAGEMENT KNOWLEDGE (not on zoological/herpetological and snake physiology knowledge, which are not very relevant for MOH activities and trainings towards saving lives and limbs.

**RESPONSE 7**

I wish to disagree with reviewer 2 and our response is same as response 6.. This was what the WHO said *“Management of snakebites at different levels of the health service: all levels of the health service can contribute to the management of patients with suspected snakebite. In rural areas, where snakebites are most common, transfer to a hospital may not be feasible, and so a lower level of health facility must cope with these medical emergencies. Training of doctors, nurses, dispensers, health assistants and paramedic in diagnosis (including snake identification), early management and indications for and practicalities of administering antivenom is essential* (pp 114 of WHO guideline on snakebite treatment, <https://apps.who.int/iris/handle/10665/249547> ) The WHO added that the most important decision in managing snakebite victims is whether or not they need antivenom. Beside the symptoms that are presented by the victim, a healthcare provider knowing that snake A is non venomous will prevent administration of antisnake venom which can on its own cause serious adverse reactions. Knowing that venom of snake B has effect on the nervous system will assist in optimal management of the snakebite. This manuscript did not however have any result on zoological/herpetological and snake physiology.

**COMMENT 8**

8. SB victims in the intro you state the WHO upper limit of estimated SB incidence (world) 139,000 and then 138,000 (the latter being the official WHO number); change 139 -138.

**RESPONSE 8**

Thanks. Well noted. The change effected as suggested

**COMMENT 9**

9. Intro and intro to discussion are a bit long, and not really to the point: Could be shorter. Small typo: Laos PDR (not Loas PDR, line 363).

**RESPONSE 9**

Name of country Laos PDR now correctly spelt. Not sure if the Introduction section is that long. It is less than a page. The introduction to the discussion was to bring to the fore the importance of a knowledgeable health worker in ensuring proper management of snakebite. The study was aimed to ascertain if the health providers were really knowledgeable about snakebite management and assess if they have not overestimated what they know.

**COMMENT 10**

10. Thanks again for building this important body of evidence on SBE and issues around clinical management.

**RESPONSE 10**

Very grateful

Reviewer 3

**COMMENT 1**

1. Study sites: it is unclear if the study sites are representative of the health facilities in the region. Are the study sites the only healthcare facilities in the region? How was the selection of the sites done? Was it done by purposive or probability sampling technique?

**RESPONSE 1**

The study sites are the major health facilities in the three (3) out of the eighteen (18) districts of the Volta region of Ghana. Purposive sampling techniques was applied. So, the 4 major hospitals were chosen since they will have all the categories of health professionals required for this study. If random sampling was applied, it was possible that the facilities chosen may not have the various categories of health care professionals. The health centres and the CHPS facilities do not have medical doctors and pharmacists. The other facilities were chosen such that they will not be so close to the major hospitals. These districts were chosen because is it located in the coastal savanna ecozone which is known to have more snake bites.

**COMMENT 2**

2. The response rate is normally presented in the first paragraph of the results section.

**RESPONSE 2**

Well noted. Response rate relocated as suggested.

**COMMENT 3**

3. The response rate was lower than anticipated (76%). This could be due to the use of google form instead of face-to-face interview. What proportion of the health workers especially those in the rural areas use mobile phones or the internet. This is a major concern.

**RESPONSE 3**

Although the response rate was lower than the expected 90%, we must say for an online survey to garner a response rate of 76% is rather commendable. Face to face interaction have its own challenges. For a questionnaire that was assessing knowledge, allowing respondents to complete the questionnaire in their own environment can be a plus. When it comes to the health workers in the study area using android phones and internet services, it should not be a problem. Please read (<https://www.myjoyonline.com/business/ghanas-mobile-phone-subscribers-to-hit-40m-by-2020-report/#:~:text=The%20report%20launched%20by%20E,a%20penetration%20rate%20of%20119%25>.)

**COMMENT 4**

4. There is a need to rephrase question #5 on table 2, i.e. “How to you triage Snakebite?”

**RESPONSE 4**

Correction effected. Thanks

**COMMENT 5**

5. Otherwise the objectives are clear, study design is appropriate, statistical analysis is appropriate, and no major ethical concerns noted.

**RESPONSE 5**

Thanks.

**COMMENT 6**

6. The analysis presented matched the analysis plan

**RESPONSE 6**

Thanks

**COMMENT 7**

7. Result are clearly presented except few typographic mistakes

**RESPONSE 7**

Thanks.

**COMMENT 8**

8. Tables are of sufficient quality

**RESPONSE 8**

Thanks

**COMMENT 9**

Discussion

1. Page 26, line 364-367: It is doubtful if fear of snakes is plausible enough to explain why female health workers in this study are less knowledgeable of snakebite management than their male counterparts.

**RESPONSE 9**

That was a plausible reason in our humble opinion. Human generally would not want to learn about thinks they fear so we postulate that fear of snakes by the females limits their knowledge about management of the consequences of snakebites. (Refer to Makashvili, M., Kaishauri, N., & Azmaiparashvili, T. (2014). The role of knowledge in overcoming snake fear. *Procedia-Social and Behavioral Sciences*, *152*, 184-187. Available at <https://core.ac.uk/reader/82562530>) Also worth reading is the publication by Alves, R. R., Silva, V. N., Trovão, D. M., Oliveira, J. V., Mourão, J. S., Dias, T. L., ... & Vieira, W. L. (2014). Students’ attitudes toward and knowledge about snakes in the semiarid region of Northeastern Brazil. *Journal of ethnobiology and ethnomedicine*, *10*(1), 1-8.

**COMMENT 10**

2. Page 28, line 404: “intravascular” should be replaced by “intravenous”.

**RESPONSE 10**

Well noted. Change effected
