# Supplementary material for: Health professionals' overestimation of knowledge on snakebite management, a threat to the survival of snakebite victims—A cross-sectional study in Ghana
Source: PLoS Negl Trop Dis. 2021 Jan 19;15(1):e0008756. doi: 10.1371/journal.pntd.0008756 (PMC7846110; doi:10.1371/journal.pntd.0008756)
Supplement: Peer review history

# Peer review: Decision Letter 0

**DOI:** 10.1371/journal.pntd.0008756.r001

**Authors:** Janaka de Silva, Eugene Valentine Erulu

28 Oct 2020

Dear Mr. Ameade,

Thank you very much for submitting your manuscript “Health professionals’ over estimation of knowledge on snakebite management, a threat to survival of snake bite victims -A cross-sectional study in Ghana.” (#PNTD-D-20-01539) for review by PLOS Neglected Diseases. Your manuscript was fully evaluated at the editorial level and by independent peer reviewers. The reviewers appreciated the attention to an important problem, but raised very substantial concerns about the manuscript as it currently stands. These concerns, given below, must be addressed fully before we would be willing to consider a revised version of your study. We cannot, of course, promise publication at that time.

We therefore ask you to modify the manuscript according to the review recommendations before we can consider your manuscript for acceptance. Your revisions should address the specific points made by each reviewer, especially those raised by Reviewers #1 and #3. Your revised manuscript is also likely to be sent to reviewers for further evaluation.

We would also like to suggest to you the use of grammarly.com; a free online resource that will assist you to correct some of the grammatical mistakes and errors in the manuscript. 

When you are ready to resubmit, please upload the following:

[1] A letter containing a detailed list of your responses to the review comments and a description of the changes you have made in the manuscript. Please note while forming your response, if your article is accepted, you may have the opportunity to make the peer review history publicly available. The record will include editor decision letters (with reviews) and your responses to reviewer comments. If eligible, we will contact you to opt in or out.

[2] Two versions of the revised manuscript: one with either highlights or tracked changes denoting where the text has been changed; the other a clean version (uploaded as the manuscript file). 

Important additional instructions are given below your reviewer comments.

Please prepare and submit your revised manuscript within 60 days. If you anticipate any delay, please let us know the expected resubmission date by replying to this email. Please note that revised manuscripts received after the 60-day due date may require evaluation and peer review similar to newly submitted manuscripts.

Thank you again for your submission. We hope that our editorial process has been constructive so far, and we welcome your feedback at any time. Please don't hesitate to contact us if you have any questions or comments.

Sincerely,

Eugene Erulu, MD

Guest Editor

PLOS Neglected Tropical Diseases

Janaka de Silva

Deputy Editor

PLOS Neglected Tropical Diseases

***********************

Reviewer 1

1. The authors' hypothesis (50% of people showing sufficient knowledge of the management of snakebite envenoming) is speculative and unfounded. 

2. The objective of the study is not clearly established. The design of the study aims only to establish a state of knowledge of an ill-defined audience. 

3. The sample size is large but the power is not determined.

4. There is no statistical analysis to relate participants' responses to their occupational category and their involvement in the management of snakebite envenoming.

5. There is no analysis plan and justification presented explaining the structure and details of the questionnaire.

6. The results are clear but presented in an incomplete way.

7. The figures (tables, images) are of sufficient quality.

8. The conclusions are trivial and limited to the objectives of the study, i.e. to show no more than insufficient knowledge of health workers about snakebite envenoming.

9. The limitations of the study and analysis are not described. The selection of participants is based on respondents to an open online survey, without control over selection by designers. Furthermore, the analysis did not establish a link between participants' responses and their status or responsibility in the management of snakebite envenoming.

10. The authors do not discuss how the data can be useful in advancing our understanding of the topic in question.

11. The relevance of public health is taken into account without comments, suggestions or recommendations for intervention based on the results.

12. The manuscript "Health professionals’ over estimation of knowledge on snakebite management, a threat to survival of snake bite victims - A cross-sectional study in Ghana" by Ameade et al. has already been submitted to PLoS NTD and recently rejected. Although the topic is very important and the authors have extensively revised the article, there are still significant flaws in the methodology, and interpretation and capitalization of the results. One of my main concerns is the choice of sets of questions between the first manuscript and the second. Essential questions disclosed in the second manuscript (tables 2 and 3) did not appear in the first and, conversely, important questions presented in the first manuscript (table 4: Knowledge of snake bite, Q21 to Q35) are not included in the second… More generally, none of the answers to the questions concerning Knowledge on management of snake bites, Q36 to Q60 appeared in the first manuscript.

13. Another major concern is the ambiguity of the objective sought by the authors. The subject is essential but its importance is linked more to the knowledge necessary for health personnel to plan their training and perform their tasks, than to the level of current knowledge. Knowing the latter is only of interest if training is carried out and a second survey assesses the acquisition of knowledge and the relevance of the training. In addition, knowledge analysis should be correlated to the category of personnel and be used as an argument for proposing appropriate solutions.

14. Finally, I do not trust surveys launched on the Internet without a clearly defined strategy, not targeting an accurate audience and without specific objectives for suitable exploitation of the results.

15. However, adding these answers and making corrections to the first manuscript does not change most of the criticisms made by reviewers on the first submission.

• The questionnaire has not been previously validated, in particular by snakebite envenoming relevant professionals.

• The calculation of the number of participants required is not very interesting compared to the selection of participants, which should be more rigorous and better documented to confirm that they are representative of the health personnel.

• The rationale for the choice of the sample (involving a priori self-responders) is not clear, nor that of the selected centers, especially in relation to their regular management of snakebites.

• The age of the respondents is less than 40 years for more than 95% of the participants, which is probably not representative of the health personnel in this region of Ghana. In addition, the participant sample lacks of more experienced people who could play a crucial role in the training of the youngest.

• The distribution of responses according to categories of health personnel is not analyzed. This is all the more regrettable asone cannot expect the same level of knowledge from all categories of health workers: more knowledge is expected from a doctor than from a nurse.

• Some relevant questions are missing such as, for example, the training received after school by the participants (Q2, table 2).

• More important, some of the answers suggested by the authors in Table 3 do not seem appropriate:

-The indications for treatment with antivenom (SAV) do not include edema or necrosis, which are possible indications.

- The first choice of drug in the management of adverse reactions caused by SAV depends on the severity of the symptoms. For pruritus, hives, or mild general disorders (malaise, nausea, and fever), adrenaline – and possibly corticosteroids - are certainly not the appropriate first-line treatment. Adrenaline should be used in case of anaphylactic shock and corticosteroids act more slowly than antihistamines, which are very effective and suitable for the treatment of moderate allergy. However, the question does not refer to the level of severity, which is essential in the event of an adverse reaction. In addition, promethazine is an antihistamine: why suggest both?

- The route of administration of first choice drug in the management of adverse reactions should be deep subcutaneous rather than intramuscular in case of hemorrhagic syndrome, which is common in snakebite envenoming in sub-Saharan Africa.

- It does not seem wise or appropriate to suggest administration of SAV "months or

even years" after expiration date. The effectiveness is probably retained long after

the expiration date, but safety is not proven.

However, a scientific journal cannot suggest violating a legal provision...

- The period a suspected snakebite victim to be detained, also depends on the time

between the bite and presentation to the hospital. In the absence of any clinical sign,

24 hours seems very long and expensive for the patient.

- At least one of the questions concerns the events between January and June, while

participants started to answer in May (lines 122 and 123), which is not consistent.

Reviewer 2

Thanks for the opportunity of reading this very interesting paper. It is a very important body of evidence, which could rapidly translate into action. 

1. The methodology is transparently explained. However, no random cluster sampling was used, a "convenience sampling" was used, and only 3 out of 61 districts were surveyed (as stated in the limitations). 

2. The main results (knowledge deficit) are however sufficiently evident.

3. Sample size calculation shows that the power expected was not fully achieved but overall the sample seems sufficient for the large group conclusions. However subgroup analyses could be biased by the lack of power (samples too small) to infer conclusions, given the diversity of health facility type/ profession type. Therefore I would address this more among the study limitations. 

4. Limitations. The chapter should be a bit longer.

5. Table 5 is slightly too complex for the non-statistician reader (I was trained so I understand but I am not sure that all details, like all means, in this table are necessary).

6. The conclusions are well supported and clear with the exception of the knowledge on snake species (photos in your google-form) which don't seem very relevant to me.

7. Health staff (nu, mw, md) do not need to know about snake recognition: knowledge should rather be based on CLINICAL MANAGEMENT KNOWLEDGE (not on zoological/herpetological and snake physiology knowledge, which are not very relevant for MOH activities and trainings towards saving lives and limbs. 

8. SB victims in the intro you state the WHO upper limit of estimated SB incidence (world) 139,000 and then 138,000 (the latter being the official WHO number); change 139 -138.

9. Intro and intro to discussion are a bit long, and not really to the point: Could be shorter. Small typo: Laos PDR (not Loas PDR, line 363).

10. Thanks again for building this important body of evidence on SBE and issues around clinical management.

Reviewer 3

1. Study sites: it is unclear if the study sites are representative of the health facilities in the region. Are the study sites the only healthcare facilities in the region? How was the selection of the sites done? Was it done by purposive or probability sampling technique?

2. The response rate is normally presented in the first paragraph of the results section.

3. The response rate was lower than anticipated (76%). This could be due to the use of google form instead of face-to-face interview. What proportion of the health workers especially those in the rural areas use mobile phones or the internet. This is a major concern.

4. There is a need to rephrase question #5 on table 2, i.e. “How to you triage Snakebite?”

5. Otherwise the objectives are clear, study design is appropriate, statistical analysis is appropriate, and no major ethical concerns noted.

6. The analysis presented matched the analysis plan

7. Result are clearly presented except few typographic mistakes

8. Tables are of sufficient quality

GENERAL COMMENTS

1. The knowledge and skills of health workers regarding snakebite management in west Africa remain an important topic that is related to the increased morbidity and mortality from snakebite envenoming in the region.

2. The study objectives are clear

3. The study design is appropriate

4. The statistical analysis is appropriate

5. There are no ethical concerns envisaged

SPECIFIC COMMENTS

Methods

1. Study sites: it is unclear if the study sites are representative of the health facilities in the region. Are the study sites the only healthcare facilities in the region? How was the selection of the sites done? Was it done by purposive or probability sampling technique?

2. The response rate is normally presented in the first paragraph of the results section.

3. The response rate was lower than anticipated (76%). This could be due to the use of google form instead of face-to-face interview. What proportion of the health workers especially those in the rural areas use mobile phones or the internet. This is a major concern.

4. There is a need to rephrase question #5 on table 2, i.e. “How to you triage snakebite?”

Discussion

1. Page 26, line 364-367: It is doubtful if fear of snakes is plausible enough to explain why female health workers in this study are less knowledgeable of snakebite management than their male counterparts.

2. Page 28, line 404: “intravascular” should be replaced by “intravenous”.

Figure Files:

While revising your submission, please upload your figure files to the Preflight Analysis and Conversion Engine (PACE) digital diagnostic tool, https://pacev2.apexcovantage.com. PACE helps ensure that figures meet PLOS requirements. To use PACE, you must first register as a user. Then, login and navigate to the UPLOAD tab, where you will find detailed instructions on how to use the tool. If you encounter any issues or have any questions when using PACE, please email us at figures@plos.org.

Data Requirements:

Please note that, as a condition of publication, PLOS' data policy requires that you make available all data used to draw the conclusions outlined in your manuscript. Data must be deposited in an appropriate repository, included within the body of the manuscript, or uploaded as supporting information. This includes all numerical values that were used to generate graphs, histograms etc.. For an example see here: http://www.plosbiology.org/article/info%3Adoi%2F10.1371%2Fjournal.pbio.1001908#s5.

Reproducibility:

To enhance the reproducibility of your results, PLOS recommends that you deposit laboratory protocols in protocols.io, where a protocol can be assigned its own identifier (DOI) such that it can be cited independently in the future. For instructions see https://journals.plos.org/plosntds/s/submission-guidelines#loc-methods

# Author comment: Author response to Decision Letter 0

**DOI:** 10.1371/journal.pntd.0008756.r002

18 Nov 2020

Attachment Submitted filename: Letter - Responses to reviewers comments.docx Click here for additional data file.

# Editor report: Decision Letter 1

**DOI:** 10.1371/journal.pntd.0008756.r003

**Authors:** Janaka de Silva, Eugene Valentine Erulu

1 Dec 2020

Dear Dr Ameade,

We are pleased to inform you that your manuscript 'Health professionals’ over estimation of knowledge on snakebite management, a threat to the survival of snakebite victims - A cross-sectional study in Ghana.' has been provisionally accepted for publication in PLOS Neglected Tropical Diseases.

Before your manuscript can be formally accepted you will need to complete some formatting changes, which you will receive in a follow up email. A member of our team will be in touch with a set of requests.

Please note that your manuscript will not be scheduled for publication until you have made the required changes, so a swift response is appreciated.

IMPORTANT: The editorial review process is now complete. PLOS will only permit corrections to spelling, formatting or significant scientific errors from this point onwards. Requests for major changes, or any which affect the scientific understanding of your work, will cause delays to the publication date of your manuscript.

Should you, your institution's press office or the journal office choose to press release your paper, you will automatically be opted out of early publication. We ask that you notify us now if you or your institution is planning to press release the article. All press must be co-ordinated with PLOS.

Thank you again for supporting Open Access publishing; we are looking forward to publishing your work in PLOS Neglected Tropical Diseases.

Best regards,

Eugene Valentine Erulu, MD

Guest Editor

PLOS Neglected Tropical Diseases

Janaka de Silva

Deputy Editor

PLOS Neglected Tropical Diseases

***********************************************************

# Editor report: Acceptance letter

**DOI:** 10.1371/journal.pntd.0008756.r004

**Authors:** Janaka de Silva, Eugene Valentine Erulu

14 Jan 2021

Dear Mr Ameade,

We are delighted to inform you that your manuscript, "Health professionals’ over estimation of knowledge on snakebite management, a threat to the survival of snakebite victims - A cross-sectional study in Ghana.," has been formally accepted for publication in PLOS Neglected Tropical Diseases.

We have now passed your article onto the PLOS Production Department who will complete the rest of the publication process. All authors will receive a confirmation email upon publication.

The corresponding author will soon be receiving a typeset proof for review, to ensure errors have not been introduced during production. Please review the PDF proof of your manuscript carefully, as this is the last chance to correct any scientific or type-setting errors. Please note that major changes, or those which affect the scientific understanding of the work, will likely cause delays to the publication date of your manuscript. Note: Proofs for Front Matter articles (Editorial, Viewpoint, Symposium, Review, etc...) are generated on a different schedule and may not be made available as quickly.

Soon after your final files are uploaded, the early version of your manuscript will be published online unless you opted out of this process. The date of the early version will be your article's publication date. The final article will be published to the same URL, and all versions of the paper will be accessible to readers.

Thank you again for supporting open-access publishing; we are looking forward to publishing your work in PLOS Neglected Tropical Diseases. 

Best regards,

Shaden Kamhawi

co-Editor-in-Chief

PLOS Neglected Tropical Diseases

Paul Brindley

co-Editor-in-Chief

PLOS Neglected Tropical Diseases
